# Validation of a Reversed-Phase High Performance Liquid Chromatography Method for the Simultaneous Analysis of Cysteine and Reduced Glutathione in Mouse Organs

**DOI:** 10.1155/2016/1746985

**Published:** 2016-01-17

**Authors:** Serena Brundu, Lucia Nencioni, Ignacio Celestino, Paolo Coluccio, Anna Teresa Palamara, Mauro Magnani, Alessandra Fraternale

**Affiliations:** ^1^Department of Biomolecular Sciences, University of Urbino “Carlo Bo”, 61029 Urbino, Italy; ^2^Department of Public Health and Infectious Diseases, Pasteur Institute, Cenci-Bolognetti Foundation, “Sapienza” University of Rome, 00161 Rome, Italy; ^3^San Raffaele Pisana Scientific Institute for Research, Hospitalization, and Health Care, 00163 Rome, Italy

## Abstract

A depletion of reduced glutathione (GSH) has been observed in pathological conditions and in aging. Measuring GSH in tissues using mouse models is an excellent way to assess GSH depletion and the potential therapeutic efficacy of drugs used to maintain and/or restore cellular redox potential. A high performance liquid chromatography (HPLC) method for the simultaneous determination of GSH and cysteine (Cys) in mouse organs was validated according to USA and European standards. The method was based on separation coupled with ultraviolet detection and precolumn derivatization with 5,5′-dithiobis-(2-nitrobenzoic acid) (DTNB). The required validation parameters, that are, selectivity, linearity, lower limit of quantification, precision, accuracy, recovery, and stability, were studied for spleen, lymph nodes, pancreas, and brain. The results showed that the lower limits of quantification were 0.313 *μ*M and 1.25 *μ*M for Cys and GSH, respectively. Intraday and interday precisions were less than 11% and 14%, respectively, for both compounds. The mean extraction recoveries of Cys and GSH from all organs were more than 93% and 86%, respectively. Moreover, the stability of both analytes during sample preparation and storage was demonstrated. The method was accurate, reliable, consistent, and reproducible and it was useful to determine Cys and GSH in the organs of different mouse strains.

## 1. Introduction

GSH is the prevalent nonprotein thiol in animal cells and the most abundant antioxidant in aerobic cells. It is implicated in many cellular functions, such as degradation and synthesis of proteins and DNA or detoxification of toxins and carcinogens [1].

A depletion or an imbalance of GSH has been observed in several pathological conditions such as neurodegenerative diseases, cystic fibrosis, viral infections, AIDS, diabetes, cancer, and ageing [2, 3]. Moreover, GSH content plays an important role in regulating cellular immune response [4]. Under conditions of moderate oxidative stress, oxidation of Cys residues can lead to the reversible formation of mixed disulfides between protein thiol groups and low-molecular-mass thiols (S-thiolation), particularly with GSH (S-glutathionylation). S-Glutathionylated proteins can be readily reduced to free thiol groups when normal cellular redox status is recovered by reducing agents [5]. Moreover, to restore GSH levels, cells can use Cys causing decrease in this amino acid content [6]. Hence, GSH and Cys could be considered important biomarkers to assess the degree of oxidative damage and the correct redox state replenishment. Development and validation of simple analytical methods to measure GSH and other thiols in biological samples are a prerequisite to obtain an accurate assessment of the degree of oxidative damage as well as indication of disease progression and consequently evaluation of the effectiveness of antioxidant therapy [5]. In fact, molecules able to augment intracellular GSH levels have been proposed as potential therapeutic tools to combat several diseases and in new immunomodulatory approaches [7]. Hence, determining thiol status is important to understand the basic biochemical response of cells during a pathological condition as well as during ageing and the capacity of drugs to restore cellular glutathione homeostasis.

A lot of methods for measuring thiol species in biological fluids and tissue samples have been evolved [8–15]. However, a few methods have been validated according to US and European standards [9, 10].

Our goal was to validate a simple and rapid method, which allowed the simultaneous determination of the main thiol species, such as GSH and Cys, in different mouse organs by using reversed-phase high performance liquid chromatography (RP-HPLC) with ultraviolet detection. The method was based on precolumn derivatization with Ellman's reagent [5,5′-dithiobis-(2-nitrobenzoic acid), (DTNB)] which reacted with R-SH to form the R-TNB derivative which was separated and quantified. Among the different available derivatization methods, the quantification of thiols by DTNB was selected because it has been described to be particularly simple and useful in the study of thiol redox state and protein glutathionylation [16]. Moreover, RP-HPLC method has the advantage of being accessible to most analytical laboratories since they do not require expensive dedicated instruments.

The method proved good in quality and performance and allowed determining GSH and Cys in the organs of three mouse strains (ICR (CD-1), BALB/cJ, and C57BL/6N mice) commonly used in preclinical studies.

## 2. Materials and Methods

### 2.1. Chemicals

Cys, GSH, and DTNB were purchased from Sigma-Aldrich (Sigma-Aldrich Co., St. Louis, MO, USA). Acetonitrile was acquired from Carlo Erba (Carlo Erba Reagenti, Milan, Italy).

### 2.2. Ethics Statement

Housing and treatment of mice were in compliance with the recommendations in the Guide for the Care and Use of Laboratory Animals by the Health Ministry, law 116, 1992. Experiments were approved by the Committee on the Ethics of Animal Experiments of the University of Urbino “Carlo Bo” and Sapienza University. The animals were suppressed by carbon dioxide. Every effort was made to minimize animal suffering and to limit the number of animals used.

### 2.3. Animals

Four-week-old female ICR (CD-1) and six-week-old female BALB/cJ mice were purchased from Harlan Nossan (Milan, Italy), while four-week-old female C57BL/6N mice were purchased from Charles River (Lecco, Italy). Throughout the study, the mice were kept at a temperature of 22 ± 1°C and a relative humidity of 60 ± 5%, with a 12 h light/dark cycle and 12 air changes/h.

### 2.4. High Performance Liquid Chromatography Apparatus

Cys and GSH determination in different organs was performed through HPLC Jasco Model LG-980-02 (Jasco Europe S.R.L., Cremella (LC), Italy). The separation was performed on a Teknokroma Tracer Excel 120 column ODSA 5 *μ*m 15 × 0.46 (Teknokroma Analitica S.A., Barcelona, Spain) protected by a Teknokroma Tracer Excel guard column ODS 10 × 3.2 mm (Teknokroma Analitica S.A., Barcelona, Spain). The mobile phase consisted of KH_2_PO_4_ solution (10 mM, pH 6.0) (buffer A) and buffer A containing acetonitrile (60% v/v) (buffer B). All buffer solutions after preparation and pH adjustment as well as standards were filtered through 0.22 *μ*m Acrodisc Syringe Filters (Pall Life Sciences, Ann Arbor, MI, USA). The elution conditions were as follows: 10 min 100% buffer A, followed by an increase to 100% buffer B in 15 min; this condition was maintained for 5 min. The gradient was returned to 100% buffer A in 3 min, and the column was regenerated with 100% buffer A for another 4 min before injection of the next sample. The flow rate was 1 mL/min, the injection volume was 50 *μ*L, and detection was at 330 nm. Analyses were performed at 25°C and quantitative measurements were obtained by injection of standards of known concentration.

### 2.5. Sample Preparation

The organs (spleen, lymph nodes, brain, and pancreas) were quickly excised at the same time of the day (9 a.m.–11 a.m.), 10–20 mgs were immediately put into an Eppendorf microcentrifuge tube containing 500 *μ*L of precipitating solution (100 mL containing 1.67 g of glacial metaphosphoric acid, 0.2 g of disodium EDTA, and 30 g of NaCl). The sample was first homogenized through a grinding pestle and then sonicated at 50 watts for 10 seconds (B. Braun Labsonic U, B. Braun Biotech International); all of these procedures were carried out in ice. The sample was kept in ice for 10 min and then centrifuged at 12,000 ×g for 10 min at 4°C. Fifteen *μ*L of 0.3 M Na_2_HPO_4_ were added to 60 *μ*L of the acid extract and immediately after 45 *μ*L DTNB were added. DTNB solution was prepared dissolving 20 mg of DTNB in 100 mL of sodium citrate solution (1% w/v). The mixture was stirred for 1 minute at room temperature (RT) and then left at RT for another 5 minutes and finally used for Cys and GSH determination by RP-HPLC.

### 2.6. Method Validation

The method was validated according to the currently accepted US-FDA Bioanalytical Method Validation Guidance and European Medicines Agency Guideline on Bioanalytical Method Validation with respect to selectivity, linearity, lower limit of quantification (LLOQ), precision and accuracy, recovery, and stability. The method was validated with organs of ICR (CD-1) mice. Selectivity was assessed by comparing chromatograms of standard preparations with those of mouse organs.

Calibration curves for GSH and Cys were obtained by serial dilutions from a stock solution. The standards were diluted either in water or in the precipitating solution used to precipitate organ proteins. The exact concentrations of standard solutions of GSH and Cys were obtained through spectrophotometer readings at 412 nm following the procedure described by Beutler [17]. The linearity of each calibration curve was determined by plotting the peak area (*y*) versus the corresponding concentration (*x*). The LLOQ was defined as the lowest calibration standard on the calibration curve with acceptable accuracy within 20% and precision below 20%.

The precision and accuracy of the method were assessed by at least five replicate analyses of organ samples spiked with GSH and Cys ranging from low to high concentrations of the calibration curve. The precision was evaluated in the same analytical run (intraday assay) or in at least five analytical days (interday assay), one of which was in the subsequent week. Precision was defined as the relative standard deviation (RSD%), while accuracy was defined as relative error (RE%), both not exceeding 15%.

Recoveries were calculated by adding known concentrations of GSH and Cys to the organ before submitting it to the processing steps of the method, and the final concentration of each sample represented the mean of five measurements. Results are provided as the difference between the measured and the theoretical values and expressed as percentage of recovery.

### 2.7. Sample Stability

Sample stability was determined by analyzing organs that were excised, processed, and left for 4 h at room temperature or for 8 h at 4°C or after three freeze-thaw cycles. Sample stability was also evaluated in organs that were excised and frozen in the precipitating solution at −80°C for 3 months. Stability sample results should be within 15% of the analyte concentration encountered in the sample immediately processed.

## 3. Results and Discussion

Selective, sensitive, and validated analytical methods for the quantitative evaluation of GSH and other thiol species are critical for determination of redox state in experimental models and the successful conduct of preclinical and/or clinical pharmacology studies employing molecules to restore GSH levels that can be altered in pathological conditions [2, 7]. Validating bioanalytical methods demonstrates that a particular method used for quantitative measurement of analytes in a given biological matrix (e.g., mouse organs) is reliable and reproducible. Fundamental parameters for this validation include the following: selectivity, sensitivity, accuracy, precision, reproducibility, and stability. Unfortunately, only a few methods for the determination of GSH and Cys in biological samples have been validated [9, 10]. In this paper, we described validation of a simple, rapid, sensitive, and cost-effective RP-HPLC method for the simultaneous determination of Cys and GSH in different mouse organs according to US and European standards, which can make it of interest to readers who have to measure tissue GSH.

### 3.1. Selectivity

**Figure 1:**
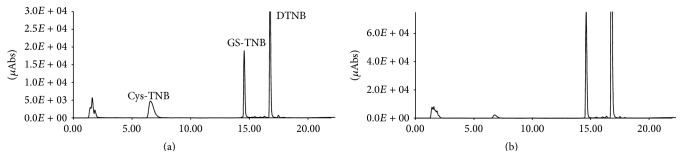
HPLC chromatograms of standards of Cys and GSH (20 *μ*M) (a) and of an extract of mouse spleen (b). The exact concentrations of standard solutions of Cys and GSH were obtained through spectrophotometer readings at 412 nm as described in Section 2.

Selectivity is the ability of an analytical method to differentiate and quantify the analyte in the presence of other components in the sample. The retention times of Cys and GSH were 6.7 ± 0.4 and 14.8 ± 0.5 min, respectively. No significant interference from endogenous substances was observed at the retention times of the compounds studied.  Figure 1 shows a representative chromatogram of GSH and Cys standards (a) and mouse spleen (b). The chromatograms of other organs were comparable to spleen chromatograms (data not shown).

### 3.2. Linearity and LLOQ

**Figure 2:**
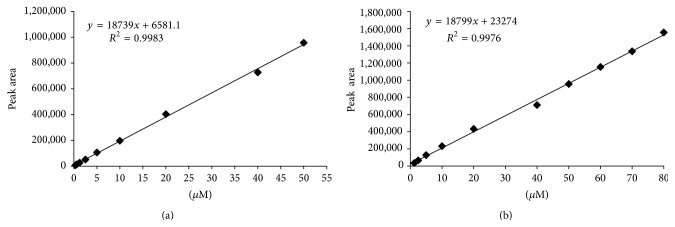
Calibration curves of Cys (a) and GSH (b) diluted in the solution used to precipitate organ proteins. Standards solutions were quantified through spectrophotometer readings at 412 nm as described in Section 2.

The standards used for the calibration curve were diluted either in water or in the precipitating solution used to precipitate the organ proteins, obtaining two comparable curves.  Figure 2 shows the typical calibration curves and linearity ranges for Cys (a) and GSH (b) diluted in the precipitating solution. The calibration curves were linear in the range of 0.313–50 *μ*M and 1.25–80 *μ*M for Cys and GSH, respectively.

The LLOQ for Cys was 0.313 *μ*M, and the precision and accuracy were less than 8% and within ±5%, respectively. The LLOQ for GSH was 1.25 *μ*M with a precision less than 11% and an accuracy lower than ±9%.

### 3.3. Sample Stability

Stability of thiol compounds during prolonged storage of the tissues in the protein precipitating buffer or of the deproteinized tissue homogenates is a prerequisite for reliable analysis in experimental setting. Stability was evaluated in samples stored in different conditions as described in Section 2. The results obtained showed that there was no significant difference in the peak areas of Cys and GSH demonstrating the high stability of these thiol species and the validity of the sample preparation protocol in preventing their conversions (Table 1). In the first column values are referred to organs left immersed in the protein precipitating solution. In the second, third, and fourth column values are referred to as deproteinized organ homogenates (RT: room temperature). Values are the mean ± SD of 5 animals per organ. Stability samples have been compared with the same samples immediately processed and analyzed.

### 3.4. Precision, Accuracy, and Recovery

The accuracy of an analytical method describes the closeness of mean test results obtained by the method to the actual concentration of the analyte, while the precision describes the closeness of individual measures of an analyte when the procedure is applied repeatedly to a single sample.

The recovery of an analyte in an assay is the detector response obtained from an amount of the analyte added to and extracted from the biological matrix (mouse organ), compared to the detector response obtained for the true concentration of the analyte in solvent.

We evaluated the precision, accuracy, and recovery of the entire method by analyzing tissue samples spiked with 40 *μ*M, 20 *μ*M, or 2.5 *μ*M of Cys or with 60 *μ*M, 20 *μ*M, or 5 *μ*M of GSH. Each concentration was tested five times, and the data of the assay are shown in Table 2. All the results of the tested samples were within 15%, meeting the acceptable criterion. In particular, in all tissue samples, the intraday precision (*n* = 5) was less than 11%, while the interday precision (*n* = 5) was less than 14% for both thiol species.

The accuracies of all the analyzed samples were less than 9% for both thiol species, except for brain spiked with 5 *μ*M of GSH in which case it was less than 14%, indicating that the developed method is accurate and reliable.

For the recovery, the concentration in the spiked samples was expressed as a percentage of the predicted concentration, which was calculated as the sum of the added concentration and the endogenous level of the analyte in the unspiked sample. The mean extraction recoveries (*n* = 5) in all organ samples were more 93% and 90% for Cys and GSH, respectively, except in the brain spiked with 5 *μ*M GSH where the recovery was more than 86%. These results indicate that the recoveries of both analytes were consistent and reproducible and, in comparison to other HPLC-UV methods used for Cys and GSH determination in biological fluids [10], a higher recovery for both thiol species was obtained. Another advantage of the method is represented by the use of DTNB as derivatizing agent requiring shorter derivatization times. Cys and GSH were also quantified in tissue specimens by a chromatographic system equipped with a fluorescent detector [9], but HPLC with a UV detector belongs to the standard instrumentation of an analytical laboratory not requiring particular expensive maintenance.

### 3.5. Determination of Cys and GSH in Organs of Different Mouse Strains

**Figure 3:**
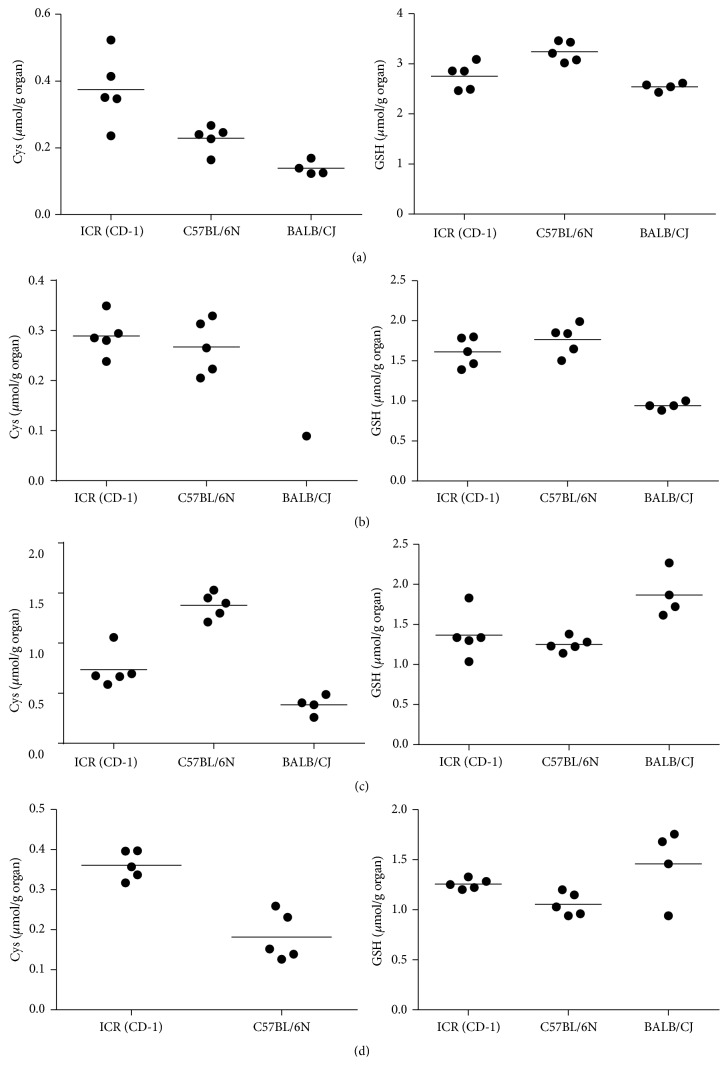
Quantification of Cys (left) and GSH (right) by HPLC in spleens (a), lymph nodes (b), pancreas (c), and brains (d) of ICR (CD-1) (*n* = 5), C57BL/6N (*n* = 5), and BALB/Cj (*n* = 4) mice.

The validated method was successfully applied to determine the concentrations of Cys and GSH in the spleen, lymph nodes, pancreas, and brain of ICR (CD-1), BALB/cJ, and C57BL/6N mice. The data reported in Figure 3 show similar content of GSH (right) in the spleen (a), pancreas (c), and brain (d) of all strains, while a lower content of GSH was found in the lymph nodes (b) of BALB/cJ. The concentration of Cys (left) found in spleen (a) and pancreas (c) was lower in this strain than that in the other ones; moreover the brain was assayed for Cys but it was not detected (d) and in only one lymph node sample (b) it was detectable. This is likely due to the lower concentration of Cys in these two organs of BALB/Cj mice; moreover, we can observe that in this strain Cys levels were lower compared to the other strains, even when measured above LLOQ. The applicability of the proposed method was assessed through the analysis of GSH and Cys in other mouse organs such as liver, kidney, lungs, and heart (Supplementary Table in the Supplementary Material available online at http://dx.doi.org/10.1155/2016/1746985) as well as the analysis of GSH-replenishing molecules containing –SH groups which were identified and clearly distinguished from GSH and Cys [18].

These data provide reliable reference measurements of GSH and Cys in the spleen, lymph nodes, pancreas, and brain of three mouse strains widely used in preclinical studies. Particularly, the data reported are a valuable resource for investigating the role of GSH in modulating several intracellular processes, from oxidative damage to immune responses, as well as the effects of drugs and toxic compounds on glutathione metabolism.

## 4. Conclusions

Results obtained from validation of the RP-HPLC method herein described show that the method is accurate, reliable, consistent, and reproducible. Moreover, the sample preparation and the extraction procedure developed allow high stability of Cys and GSH in the samples preventing their conversions. The main advantages of the present method are validation, high recovery, simplicity, short derivatization times, and low analytical costs. Therefore, this method is particularly suitable for reliable routine measurement of thiols in mouse organs and it can be used in all of those animal models mimicking human diseases characterized by GSH imbalance to both study disease processes and develop therapies including GSH-based antioxidant treatment.

## Supplementary Material

The validated method was applied to determine the concentrations of Cys and GSH in liver, kidney, lungs and heart of C57BL/6 mice. Organs were processed as described in Section 2. Then GSH and Cys were quantified through RP-HPLC method as described in Section 2.

## Figures and Tables

**Table 1:** Stability of Cys and GSH in mouse organs.

Sample	−80°C 3 months	RT 4 h	4°C 8 h	Three freeze-thaw cycles
Cys				
Spleen	101.1 ± 4.2	97.6 ± 3.0	99.3 ± 2.9	98.7 ± 1.0
Lymph nodes	97.8 ± 4.6	98.7 ± 7.8	99.8 ± 1.9	98.4 ± 3.9
Pancreas	97.6 ± 2.2	103.0 ± 3.2	96.9 ± 1.8	98.9 ± 1.4
Brain	99.1 ± 2.3	98.4 ± 1.4	99.0 ± 5.1	98.5 ± 3.9
GSH				
Spleen	99.9 ± 2.4	97.7 ± 1.7	98.9 ± 2.9	99.2 ± 1.9
Lymph nodes	98.9 ± 1.5	97.9 ± 2.3	99.1 ± 6.9	100.1 ± 1.9
Pancreas	99.3 ± 4.0	97.9 ± 2.0	98.0 ± 3.5	98.4 ± 4.1
Brain	99.6 ± 2.4	99.9 ± 7.5	99.1 ± 7.3	98.7 ± 1.4

**Table 2:** Summary of precision, accuracy, and recovery of the assay for GSH and Cys in mouse spleens, lymph nodes, pancreas, and brains (*n* = 5).

Sample	Concentration (*μ*M)	Intraday (RSD%)	Interday (RSD%)	Accuracy (RE%)	Recovery (%, mean ± SD)
Cys					
Spleen	2.5	4.6	11.7	3.8	98.4 ± 1.3
	20	5.3	8.0	5.8	96.8 ± 2.4
	40	4.6	3.9	2.6	97.7 ± 2.1
Lymph nodes	2.5	6.9	8.9	5.1	97.0 ± 2.3
	20	9.0	12.9	4.8	96.8 ± 2.6
	40	5.0	11.7	6.2	93.9 ± 5.7
Pancreas	2.5	9.1	10.7	4.3	94.2 ± 5.6
	20	7.5	6.8	8.5	94.5 ± 4.2
	40	6.2	9.9	5.7	95.6 ± 2.7
Brain	2.5	9.7	6.7	4.6	95.9 ± 2.6
	20	10.3	10.6	3.4	94.5 ± 4.0
	40	8.7	11.8	6.7	96.4 ± 2.8
GSH					
Spleen	5	3.3	7.5	3.5	92.7 ± 2.2
	20	10.8	9.7	3.3	93.5 ± 4.6
	60	9.0	12.4	1.9	94.7 ± 5.2
Lymph nodes	5	10.8	12.4	8.9	90.4 ± 2.0
	20	1.9	6.2	3.7	96.4 ± 1.5
	60	1.5	9.7	4.5	93.7 ± 4.8
Pancreas	5	9.9	12.8	3.3	91.4 ± 2.8
	20	7.5	9.4	5.0	97.4 ± 3.2
	60	9.3	13.7	6.4	91.1 ± 6.3
Brain	5	2.6	12.6	13.7	86.5 ± 5.0
	20	4.4	10.7	1.2	98.2 ± 3.7
	60	9.0	8.1	5.3	94.3 ± 7.2

## References

[1] Forman H. J., Zhang H., Rinna A. (2009). Glutathione: overview of its protective roles, measurement, and biosynthesis. *Molecular Aspects of Medicine*.

[2] Townsend D. M., Tew K. D., Tapiero H. (2003). The importance of glutathione in human disease. *Biomedicine & Pharmacotherapy*.

[3] Finkel T., Holbrook N. J. (2000). Oxidants, oxidative stress and the biology of ageing. *Nature*.

[4] Ghezzi P. (2011). Role of glutathione in immunity and inflammation in the lung. *International Journal of General Medicine*.

[5] Dalle-Donne I., Rossi R., Colombo R., Giustarini D., Milzani A. (2006). Biomarkers of oxidative damage in human disease. *Clinical Chemistry*.

[6] Lu S. C. (1998). Regulation of hepatic glutathione synthesis. *Seminars in Liver Disease*.

[7] Fraternale A., Paoletti M. F., Casabianca A. (2006). Antiviral and immunomodulatory properties of new pro-glutathione (GSH) molecules. *Current Medicinal Chemistry*.

[8] Isokawa M., Kanamori T., Funatsu T., Tsunoda M. (2014). Analytical methods involving separation techniques for determination of low-molecular-weight biothiols in human plasma and blood. *Journal of Chromatography B: Analytical Technologies in the Biomedical and Life Sciences*.

[9] Kulm K. S., Krasselt A. I., Fürst P. (2000). Glutathione and glutathione metabolites in small tissue samples and mucosal biopsies. *Clinical Chemistry*.

[10] Zhang W., Li P., Geng Q., Duan Y., Guo M., Cao Y. (2014). Simultaneous determination of glutathione, cysteine, homocysteine, and cysteinylglycine in biological fluids by ion-pairing high-performance liquid chromatography coupled with precolumn derivatization. *Journal of Agricultural and Food Chemistry*.

[11] Küster A., Tea I., Sweeten S., Rozé J.-C., Robins R. J., Darmaun D. (2008). Simultaneous determination of glutathione and cysteine concentrations and 2H enrichments in microvolumes of neonatal blood using gas chromatography-mass spectrometry. *Analytical and Bioanalytical Chemistry*.

[12] Wang W., Li L., Liu S., Ma C., Zhang S. (2008). Determination of physiological thiols by electrochemical detection with piazselenole and its application in rat breast cancer cells 4T-1. *Journal of the American Chemical Society*.

[13] Chang C.-W., Tseng W.-L. (2010). Gold nanoparticle extraction followed by capillary electrophoresis to determine the total, free, and protein-bound aminothiols in plasma. *Analytical Chemistry*.

[14] Zuo Q.-P., Li B., Pei Q., Li Z., Liu S.-K. (2010). A highly selective fluorescent probe for detection of biological samples thiol and its application in living cells. *Journal of Fluorescence*.

[15] Huang Y.-Q., Ruan G.-D., Liu J.-Q., Gao Q., Feng Y.-Q. (2011). Use of isotope differential derivatization for simultaneous determination of thiols and oxidized thiols by liquid chromatography tandem mass spectrometry. *Analytical Biochemistry*.

[16] Özyürek M., Baki S., Güngör N., Çelik S. E., Güçlü K., Apak R. (2012). Determination of biothiols by a novel on-line HPLC-DTNB assay with post-column detection. *Analytica Chimica Acta*.

[17] Beutler E. (1984). Reduced glutathione. *Red Cell Metabolism. A Manual of Biochemical Methods*.

[18] Fraternale A., Crinelli R., Casabianca A. (2013). Molecules altering the intracellular thiol content modulate NF-kB and STAT-1/IRF-1 signalling pathways and IL-12 p40 and IL-27 p28 production in murine macrophages. *PLoS ONE*.

