# Supplementary material for: Validation of a Reversed-Phase High Performance Liquid Chromatography Method for the Simultaneous Analysis of Cysteine and Reduced Glutathione in Mouse Organs
Source: Oxid Med Cell Longev. 2016 Jan 17;2016:1746985. doi: 10.1155/2016/1746985 (PMC4739232; doi:10.1155/2016/1746985)
Supplement: Supplementary file 1 — The validated method was applied to determine the concentrations of Cys and GSH in liver, kidney, lungs and heart of C57BL/6 mice. Organs were processed as described in Section 2. Then GSH and Cys were quantified through RP-HPLC method as described in Section 2. [file 1746985.f1.docx]

Supplementary table.

**Cysteine and GSH content in C57BL/6 organs (n=3).**

|  | GSH (µmoles/g organ) | Cys (µmoles/g organ) |
| --- | --- | --- |
| Liver | 7.560 ± 0.958 | 0.192 ± 0.085 |
| Kidney | 1.574 ± 0.188 | 0.401 ± 0.166 |
| Lungs | 2.112 ± 0.210 | 0.067 ± 0.018 |
| Heart | 1.018 ± 0.051 | 0.054 ± 0.016 |

The values represent the mean ± SD.
